# Effectiveness and safety of warm needle acupuncture on insomnia in climacteric women: Protocol for a systematic review and meta-analysis

**DOI:** 10.1097/MD.0000000000015637

**Published:** 2019-05-17

**Authors:** Hong Wei Xu, Wei Du, Lingling He, Xiuying Kuang

**Affiliations:** a Guangzhou University of Chinese Medicine; b The First Affiliated Hospital of Guangzhou University of Chinese, Medicine, Guangzhou, China.

**Keywords:** climacteric women, insomnia, protocol, warm needle acupuncture

## Abstract

**Background:**

Warm needle acupuncture (WNA) is a traditional Chinese medicine (TCM) therapy which combines technical advantages of acupuncture and moxibustion. Climacteric insomnia is a common symptom in climacteric women, which can seriously affect the physical and mental health of patients. Relevant studies have been reported that WNA can improve insomnia in climacteric women. In this protocol, the effectiveness and safety of WNA on insomnia in climacteric women will be explored.

**Methods:**

Seven electronic databases include 3 English databases [Excerpta Medica database (EMBASE), PubMed, the Cochrane Central Register of Controlled Trials (Cochrane Library)] and 4 Chinese databases [Chinese VIP Information, Chinese National Knowledge Infrastructure (CNKI), Chinese Biomedical Literature Database (CBM) and Wanfang Database] for randomised controlled trials (RCT) of WNA on insomnia in climacteric women will be searched. The changes of the Pittsburgh sleep quality index was used as the main outcome, and the secondary outcome includes the changes of the Kupperman score, serum hormone level, and TCM syndrome score, as well as the adverse events caused by WNA. We will use RevMan software V5.3 to help us to analyze all data and use a Cochrane risk of bias tool to help us to assess the methodological quality for RCTs.

**Result:**

This study will provide reliable evidence for WNA on insomnia in climacteric women

**Conclusion:**

The findings will be an available reference to evaluate the effectiveness and safety of WNA on insomnia in climacteric women.

**Registration:**

PROS-PERO CRD42019125743.

## 1 Introduction

### 1.1 Description of the condition

Caused by the decline of sex hormones, approximately two-thirds of climacteric women suffer from perimenopausal syndrome which characterized by disorders of endocrine system and autonomic nervous system.^[1]^ Symptoms including insomnia, great fluctuation of emotion, palpitations, and so on. The 56.6% of 6079 women aged 40 to 59 complaint about insomnia or unsatisfaction of sleep quality, according to a research.^[2]^ Besides, the incidence of insomnia increased with age and could cause serious adverse effects on health and daily life. Long-term insomnia may also lead to mental diseases which could aggravate insomnia, thus forming a vicious circle. What's more, it is reported that climacteric women with insomnia were at higher risk of death from clinical accidents caused by cardiovascular and cerebrovascular diseases.^[3]^ Therefore, it is important to improve insomnia condition of climacteric women.

### 1.2 Description of the intervention

As a common therapy and an important element of traditional Chinese medicine (TCM), warm needle acupuncture (WNA) combines technical advantages of acupuncture and moxibustion which is an effective ‘safe’ convenient treatment with almost no harmful side effects^[4]^ to heat and stimulate acupoints.^[5]^ According to literatures, WNA can enhance immunity, improve blood circulation, and prevent diseases.^[5]^ Recently, studies have found that WNA might have efficacy on insomnia in climacteric women. However, relationships between climacteric insomnia and WNA have not been clarified. Therefore, it is necessary to perform a systematic review and meta-analysis in order to explore the effectiveness and safety of WNA for insomnia in climacteric women.

## 2 Methods

### 2.1 Study registration

The protocol has been registered on the International Prospective Register of Systematic Reviews (PROSPERO) (registration number, CRD42019125743) basing on the Preferred Reporting Items for Systematic Reviews and Meta-Analyses Protocols (PRISMA-P) statement guidelines.

### 2.2 Eligibility criteria

#### 2.2.1 Research type

All randomized controlled trials (RCTs) that estimated the effectiveness and safety of WNA on insomnia in climacteric women will be collected. There is no unified requirement on the blinding and language of the findings.

#### 2.2.2 Participant type

As to the patients in the study, they are diagnosed as menopausal insomnia by clinicians based on The Chinese Classification and Diagnosis of Mental Diseases-3rd edition (CCMD-3)^[6]^ and The Clinical guideline of new drugs for TCM-3rd edition.^[7]^ In terms of gender, it has to be for women. Patients need to be between 40 and 60 years old.

#### 2.2.3 Intervention measures

Intervention group will be treated with WNA regardless of the form, type of needle, length of needle, which can combine other conventional treatments; while control group will only include only other conventional treatments.

#### 2.2.4 Outcome measures

The primary outcome measures will be as follows: changes in the Pittsburgh Sleep Quality Index (PSQI). The secondary outcome measures will include the changes of the Kupperman score^[8]^, serum hormone level and TCM syndrome score.

#### 2.2.5 Exclusion criteria

If the following situations occur in the study (repeated test and no test data required by this program are available, acquired study will be excluded.

### 2.3 Data sources

#### 2.3.1 Electronic search

Some databases such as Cochrane Library, Embase, PubMed, Chinese Scientific Journal Database (VIP), Chinese National Knowledge Infrastructure (CNKI), Chinese Biomedical and Literature Database (CBM), and Wanfang Database are helpful to identify relevant RCTs which will be searched from inception to Feb. 10th, 2019. The retrieval type will be “warm needle acupuncture” or “needle warming moxibustion” AND “insomnia in climacteric women” or “Perimenopausal insomnia”. Above terms will be translated into Chinese, which can help to search relevant findings in the Chinese databases. Endnote software 8.1 will be the reliable tool used to exclude the duplicate articles.

#### 2.3.2 Additional search

To ensure comprehensiveness of this research, we will retrieve other potential articles in the reference list of retrieved studies. As to articles not included in the electronic database or related papers and journal, further consultation will be needed.

### 2.4 Study selection and Data extraction

**Figure 1:**
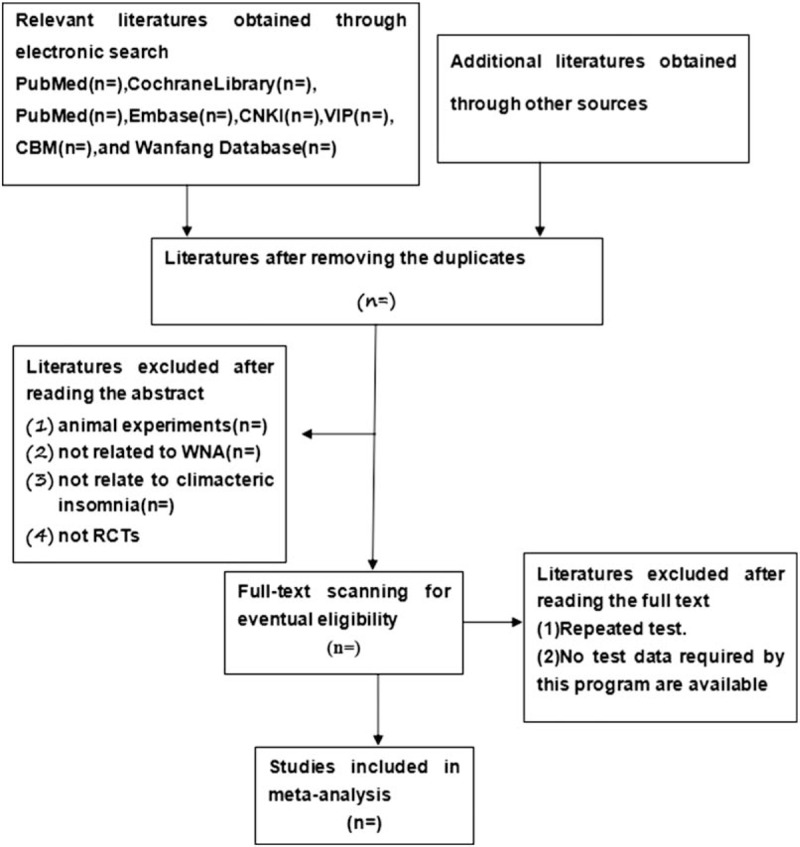
Flow diagram of study selection process.

Two reviewers will firstly adopt Endnote software 8.1 to acquire useful findings by reading abstracts of articles obtained from databases mentioned above. Then we will read through the whole findings to decide the final eligibility as a second analysis. Disagreements will be solved by discussing with other researchers. In order to make clear the study selection procedure, (Fig. 1) a Preferred Reporting Items for Systematic Reviews and Meta-Analyses (PRISMA)^[9]^ flow chart is shown. (Fig. 1)

We will extract following data: ① basic information of the included findings (e.g., author, publication year, and etc); ② essential information of the included participants (e.g., number of participants, gender, mean and standard deviation for age, and etc); ③ interventions in control group and treatment group; ④ random method; ⑤ outcome measures;

### 2.5 Data analysis

#### 2.5.1 Risk of bias in the included studies

We will use the Cochrane Handbook, version 5.1.0 to assess the risk of bias of each included RCT, which includes random sequence generation, allocation concealment, blinding of outcome assessments, incomplete outcome data and selective outcome reporting and so on. The result after the evaluation of the risk of bias in each RCT is low, high, or unclear. Two reviewers will independently perform evaluations of the methodological quality of each included study, and discuss when meets discrepancies. The quality of the inclusions will be estimated according to the modified Jadad rating scale.

#### 2.5.2 Statistical analysis

Meta-analysis and test sequential analysis (TSA) of the included studies will be handled respectively by Statistical software (RevMan software V5.3). As to evaluating the continuous variables, we choose mean difference (MD) and 95% confidence intervals (CIs). When it comes to dichotomous variables, we adapt rate ratio and 95% confidence intervals (CIs)which used to evaluate the extracted data.

#### 2.5.3 Addressing missing data

If any data important for evaluation is missed, first we will try to contact the corresponding authors of articles through some reliable ways. If the sufficient data fail to be obtained after contacting the author, we will make an analysis with the available data and discuss possible impact of missing data.

#### 2.5.4 Data synthesis

With RevMan software V5.3, we can analyze all useful data based on the results of heterogeneity test, we will choose Fixed-effect model analysis if there exists small or medium heterogeneity (I^2^ <50%); but we will choose a random-effect model analysis if heterogeneity is significant (I^2^ >50%).

#### 2.5.5 Assessment of heterogeneity

We choose I^2^ test statistics to assess the heterogeneity of included studies. If I^2^ <25%, it means no significant heterogeneity, if I^2^ = 25% to 50%, it means considered moderate heterogeneity and if I^2^ >50%, it indicates strong heterogeneity. Fixed-effect model will be conducted if the heterogeneity in trials is significant (I^2^ ≥50%). If I^2^ <50%, it represents low heterogeneity, and random-effects model will be conducted.

#### 2.5.6 Assessment of publication bias

Assuming that more than 10 RCTs are included, publication bias needs to be assessed by Begg funnel plot and Egger test. If an asymmetrical funnel plot or a *P* value of <.1 on Egger test, it will be thought to indicate the presence of publication bias. On the contrary, it means no publication bias if the points become symmetrically distributed on either side of the funnel plot.

#### 2.5.7 Subgroup analysis

As to age, interventions, controls, and population area, Subgroup analyses will be conducted, if heterogeneity is ≥50%, it means significant.

#### 2.5.8 Sensitivity analysis

If the heterogeneity is high, we will conduct sensitivity analyses based on study type, sample size, and methodological quality.

#### 2.5.9 Evidence synthesis

We will evaluate the quality of evidence of the included studies on the basis of guidelines of the GRADE (Grading of Recommendations, Assessment, Development, and Evaluation). The evidence quality will be ranked by 4 levels: high, moderate, low, or very low.

#### 2.5.10 Ethics and dissemination

Ethical approval will not be applied for because of the relevant data we extracted which does not involve any individual privacy. We will publish this research, which evaluate the effectiveness and safety of WNA on insomnia in climacteric women, in a peer-reviewed journal or conference presentations.

## 3 Discussion

At present, sedative-hypnoticin is preferred for mood fluctuation and mental illnesses, such as depression, are very common in climacteric women with insomnia. In addition, insomnia in climacteric period is closely related to the decrease of sex hormones, particulary estrogen. Estrogen affects the quality of sleep through influencing regulation of body temperature, circadian rhythm, and stress response.^[10]^ Because oral hormone drugs can improve climacteric insomnia by maintaining hormone level and treating disorders of endocrine system, hormone replacement therapy is also adapted clinically. However, the long-term use of sedatives can lead to drug dependence and hormone replacement therapy may increases risks at estrogen-related tumors such as endometrial cancer and breast cancer in climacteric women with insomnia. Thus, it is of importance to search complementary and alternative therapies with less side effects.

As far as we know, it will be the first attempt to perform a systematic review and meta-analysis of the effectiveness and safety of WNA on insomnia in climacteric women. This protocol will provide referable evidence of the efficacy and safety of WNA on insomnia in climacteric women. In addition, the protocol will also provide clinical outcome measures, therapeutic effect, adverse reactions and side effects of WNA. What is more, results will be an available reference to clinicians in clinical decision-making.

## Author contributions

Xiuying Xiu conceived the study idea. Hongwei Xu was responsible for the design of this systematic review. Wei Du contributed to the data analysis plan. Lingling He drafted the manuscript and Xiuying Xiu edited. All authors provided feedback and approved the final manuscript.

**Conceptualization:** Xiuying Kuang.

**Formal analysis:** Hong wei Xu.

**Methodology:** Wei Du.

**Writing – original draft:** Wei Du.

**Writing – review & editing:** Lingling He, Xiuying Kuang.
